# Public-Health-Driven Microfluidic Technologies: From Separation to Detection

**DOI:** 10.3390/mi12040391

**Published:** 2021-04-02

**Authors:** Xiangzhi Zhang, Xiawei Xu, Jing Wang, Chengbo Wang, Yuying Yan, Aiguo Wu, Yong Ren

**Affiliations:** 1 Research Group for Fluids and Thermal Engineering, University of Nottingham Ningbo China, Ningbo 315100, China; xiangzhi.zhang2@nottingham.edu.cn; 2 Department of Mechanical, Materials and Manufacturing Engineering, University of Nottingham Ningbo China, Ningbo 315100, China; xiawei.xu@nottingham.edu.cn; 3 Cixi Institute of Biomedical Engineering, CAS Key Laboratory of Magnetic Materials and Devices & Key Laboratory of Additive Manufacturing Materials of Zhejiang Province, Ningbo Institute of Materials Technology and Engineering, Chinese Academy of Sciences, Ningbo 315201, China; aiguo@nimte.ac.cn; 4 Department of Electrical and Electronic Engineering, University of Nottingham Ningbo China, Ningbo 315100, China; jing.wang@nottingham.edu.cn (J.W.); chengbo.wang@nottingham.edu.cn (C.W.); 5 Research Group for Fluids and Thermal Engineering, University of Nottingham, Nottingham NG7 2RD, UK; yuying.yan@nottingham.ac.uk; 6 Key Laboratory of Carbonaceous Wastes Processing and Process Intensification Research of Zhejiang Province, University of Nottingham Ningbo China, Ningbo 315100, China

**Keywords:** microfluidic system, lab-on-a-chip, separation, detection, public health

## Abstract

Separation and detection are ubiquitous in our daily life and they are two of the most important steps toward practical biomedical diagnostics and industrial applications. A deep understanding of working principles and examples of separation and detection enables a plethora of applications from blood test and air/water quality monitoring to food safety and biosecurity; none of which are irrelevant to public health. Microfluidics can separate and detect various particles/aerosols as well as cells/viruses in a cost-effective and easy-to-operate manner. There are a number of papers reviewing microfluidic separation and detection, but to the best of our knowledge, the two topics are normally reviewed separately. In fact, these two themes are closely related with each other from the perspectives of public health: understanding separation or sorting technique will lead to the development of new detection methods, thereby providing new paths to guide the separation routes. Therefore, the purpose of this review paper is two-fold: reporting the latest developments in the application of microfluidics for separation and outlining the emerging research in microfluidic detection. The dominating microfluidics-based passive separation methods and detection methods are discussed, along with the future perspectives and challenges being discussed. Our work inspires novel development of separation and detection methods for the benefits of public health.

## 1. Introduction

Public health is closely related to human wellbeing at diverse levels from our neighbor community to the national or even global security, covering the prevention, control, and treatment of major diseases, especially infectious diseases and noncommunicable chronic diseases, as well as supervision and control of food, drug, and public environmental sanitation. The infectious diseases include avian influenza, influenza, mad cow disease, an acquired immunodeficiency syndrome (AIDS), severe acute respiratory syndrome (SARS), and dengue fever, while noncommunicable chronic diseases include cancer, diabetes, and hypertension. When an infectious disease affects a large geographical area, it may cause death, destroy cities, politics, countries, disintegrate civilization, and even annihilate ethnic groups and species [1,2,3,4]. For example, the influenza pandemic claimed a high death toll in 1918, and SARS transmitted from bat broke out in 2002, affecting public health seriously [5,6]. Recently, SARS-CoV-2 virus has caused the unprecedented COVID-19 pandemic to occur and spread rapidly all over the world since December 2019 [7]. Up to 25 February 2021, there have been 111,999,954 confirmed cases and 2,486,679 confirmed death of COVID-19 around the world, posing a great threat to human health [8]. All these infectious diseases severely impact the development of the local economy and social stability. Infectious diseases can spread through air transmission, water transmission, food transmission, contact transmission, soil transmission, vertical transmission, body fluid transmission, and fecal oral transmission. Each infectious disease is caused by its specific pathogen, including viruses, bacteria, fungi, or parasites [9,10,11,12]. Based on the necessary conditions for infectious diseases such as the infection source, transmission route, and susceptible populations, three strategies can be deployed to manage infectious diseases via controlling the source of infection, cutting off the transmission routes, and isolating the susceptible populations, respectively. From the perspective of patients, the key lies in early detection, early diagnosis, early report, and early isolation. There are two main diagnostic targets for infectious diseases: pathogens or a specific antigen, antibody, or nucleic acid of an infectious pathogen [13,14,15,16]. Some of the techniques are time-consuming, labor-intensive, expensive, and unable to be carried out on-site detection because the use of bulky instruments is inevitable, which thereby hinders their applications and makes them insufficient to achieve rapid, accurate, and on-site diagnosis during a pandemic, especially in the most common and serious resource-poor areas [17,18].

In addition to infectious diseases, noncommunicable chronic diseases are also an important threat to human health, such as cardiovascular and cerebrovascular diseases, cancer, chronic respiratory diseases, and diabetes, which are mainly caused by unhealthy lifestyle and living environment. These kinds of diseases have a high incidence rate, disability rate, mortality rate, and medical expense, which can be thawed by early diagnosis and treatment. The common diagnostic methods in clinic for noncommunicable chronic diseases are tissue biopsy and liquid biopsy [19]. However, tissue biopsy is limited by sampling bias, sampling difficulty for deep tissue, and harm to patients, while liquid biopsy presents the challenges of a few samples, complex background, and gene typing polymorphism.

The health safety of food, drug, and public environmental sanitation has become a global question, such as excessive content of metals and additives, pesticide residues, and microbial contamination in food, water, gas, and soil. In the last few years, food safety accidents have occurred repeatedly [20,21]. Improved food safety analysis and testing are needed to control food contamination [22]. However, the traditional detection technology based on instrumental analysis has the disadvantages of expensive instruments, long cycle, large material consumption, complex operation, and low sensitivity, which cannot satisfy the demand of on-site, real-time, fast, and portable detection of food [23,24,25]. Meanwhile, with the increase in environment pollution, related detection, monitoring, and cleanup technologies should be developed to detect and collect toxic wastes and pollutions [26,27].

In the past decade, microfluidic technology has developed rapidly and microfluidics can lead to the combination of the sample pretreatment, separation, and detection processes into a small chip to realize the miniaturized, automated, and multifunction integrated analysis system, which find wide applications in molecular/cell biology, chemical/gene analysis, medicine, food safety, environment sensing, and other fields, because of the advantages such as less sample consumption, fast detection speed, facile operation, multifunctional integration, small size, and portability [28,29,30]. Among the numerous applications, microfluidic sensors have been developed to detect toxic gases in industrial wastewater, such as drinking water, heavy metals, and other waterborne pathogens. Microfluidic chip technology can be further integrated with electrochemical techniques, optical techniques, magnetic techniques, mass spectrometry, and other techniques to realize the separation and detection of targeted samples [31,32,33].

There are several reviews that focus on the application of microfluidic technologies in disease detection, food safety analysis, or environmental monitoring and detection. Nevertheless, there are inadequate studies focusing on unveiling the connection of microfluidics with public health, which has been arising as a global issue especially given the present COVID-19 crisis sweeping across the world. For example, it is helpful for determining infection risks to understand aerosol concentrations and persistence in public spaces because they play an important role in coronavirus transmission. However, it is difficult to measure the concentrations, which requires specialized equipment. The challenge may be tackled using microfluidics by taking advantaging of their high throughput capability and high integration level. Thanks to the advances in microfluidic development for cell separation and detection, point-of-care diagnostics are allowed, and monitoring of individual health conditions at home is possible, which greatly eases the public healthcare burden. The present study aims to give an overview of state-of-art microfluidic separation and detection technologies from the perspectives of public health, and we focus on separation and detection because they are two of the most important steps toward practical applications in disease detection, food safety analysis, and environmental monitoring and detection. The reviewed topics are closely associated with public health based on three aspects: (1) Prevention and early monitor of infectious diseases such as detection of COVID-19 viruses necessitates the demand to apply the microfluidics-based separation and detection methods; (2) Microfluidics also inspires novel routes to develop the vaccine products to effectively treat the diseases which may result in big public health impacts; and (3) The rapid growth of microfluidics-based separation and detection technologies also leads to point-of-care diagnosis which enables people to monitor their health conditions using portable devices at home, and this significantly mitigates the needs to seek medical assistance at hospitals and therefore promotes the public health level. The review paper is structured as follows: first, various microfluidic separation methods for public health are summarized and discussed. Subsequently, microfluidic detection methods applied to public health are systematically presented. Finally, the challenges and prospects of microfluidic separation and detection technology are discussed.

## 2. Microfluidic Separation Methods

Microfluidics technology is an interdisciplinary subject with many applications in various fields, such as biomedical, chemistry, disease diagnosis, and electronics industry [34]. Microfluidic devices have key functions in biomedical research, such as sample pretreatment, fluid processing, biosensing, separation and monitoring, and signal detection [35]. Among them, the microfluidic separation and classification of biological targets is quite essential for biological analysis and clinical diagnosis [36], which can be achieved with lab-on-a-chip (LOC), micrototal analytic systems (μTAS), and point-of-care (POC) diagnostics [37]. Although the development of microfluidic technology is still in its early stage, it has the potential to affect many fields from chemical synthesis and biological analysis to the disciplines of optics and information technology [38]. Microfluidic devices are able to create dynamic environments where the gradient of physiological conditions (such as pressure, temperature, and flow rate) can be kept constant, which have a low regent consumption and realize the quantitative assessment of cell migration [39]. For instance, the separation of cells to determine the content of biological molecules such as DNA, RNA, proteins, and lipids is essential in cell biology research, as well as diagnostic and therapeutic methods [40]. In the diagnosis of anemia, sorting and counting of red blood cells (RBCs) is of great importance [41]; in the diagnosis and treatment of HIV disease, the separation of CD4+ T cells from whole blood cells is essential [42]; and isolation of circulating tumor cells (CTCs) from blood cells is important for early diagnosis of cancer [43]. Microfluidic separation is also applied on the screening of cells, which is important in the detection of cancer cells [44,45]. The microfluidic separation of cells is based on their differences in physical properties [46]. When identifying CTCs, different cancer cells of epithelial origin need to be separated [47]. Suresh et al. investigated connections between single-cell mechanical properties and subcellular structural reorganization from biochemical factors in the context of gastrointestinal tumor and malaria [48]. It was found that cancer cells have larger sizes and higher deformability compared with healthy cells [49]. The deformability difference of normal red cells and red cells infected with malarial parasites can explain the mechanism of the spleen to remove parasitized red cells from the circulation of hosts [50]. In the past few decades, various separation and sorting methods have been developed for the separation of cells. Microfluidic separation and sorting has many advantages, including decreasing sample volumes, speeding up sample processing, enhancing sensitivity and spatial resolution, reducing device cost, increasing portability [51], reducing processing cost [52], raising efficiency [53], and contributing to environmental compatibility [54]. The application of polymer materials in microfluidic devices fabrication provides simple, cost-effective, and disposal advantages [55]. In order to avoid sample pollution by using biochemical markers, microfluidic techniques for label-free differentiation and fractional of cell population have been developed [40]. Droplets often act as microreactors for encapsulation. Since it can be important to ensure the droplets contain precise volume and composition or to ensure uniformity of emulsions, the separation and sorting of droplets should be taken in consideration, which can be realized by microfluidic approaches [56].

Microfluidics can be divided into two categories based on the scale: continuous microfluidics and digital microfluidics, [57,58,59]. Microparticle separation can be categorized as active and passive methods based on their manipulating forces [60]. In passive techniques, microfluidic devices do not use external forces for sorting or separation but rely purely on microfluidic phenomena and the interaction of the fluid with the geometrics of the microfluidic devices [61], while active sorting techniques involve an external field [62]. By comparing the advantages and disadvantages of passive and active techniques, Sajeesh and Kumar [36] concluded passive techniques are preferred in applications where energy input is of critical concern, whereas active separation techniques are preferred where higher particle sorting efficiency is required. The recent advances in separation and detection of whole-blood components were reviewed by Doddabasavana et al. [63]. The performance of microfluidic separation is evaluated according to the separation time, separation efficiency, throughput rate, and clogging filtration. According to separation approaches, separation techniques can be divided into passive and active methods [64]. The present paper focuses solely on passive separation/sorting approaches because they are easier to implement and thus can find more applications for public health, especially in developing countries or regions where people have limited access to costly apparatuses to energize the active separation approaches.

### 2.1. Pinched-Flow Fractionation (PFF)

The continuous sizing of particles in a microchannel is based on the characteristics of the laminar flow profile [65], and complicated outer field control is eliminated, which is usually required for other kinds of particle separation methods. Therefore, PFF can be applied both for particle analysis and for the preparation of monodispersed particles where energy input is of critical concern. The separation resolution in PFF is a function of the microchannel aspect ratio, particle size difference, and the microchannel sidewall roughness.

The work of Jain et al. [66] showed that particles with diameters on the order of the sidewall roughness cannot be separated in PFF devices with symmetric channels due to the same resistance in all outlet channels. Ma et al. [67] investigated the separation performance of an as PFF device by employing an immersed boundary-lattice Boltzmann method (IB-LBM), and the results showed that an adaptive regulating flux can be determined for each case to sort the cell mixture effectively. Yanai et al. [68] proposed a new hydrodynamic mechanism of particle separation in asPFF microchannel networks based on three-dimensional (3D) laminar flow profiles formed at intersections of lattice channels, and they confirmed that the depth of the main channel was critical for the particle separation efficiencies.

**Figure 1:**
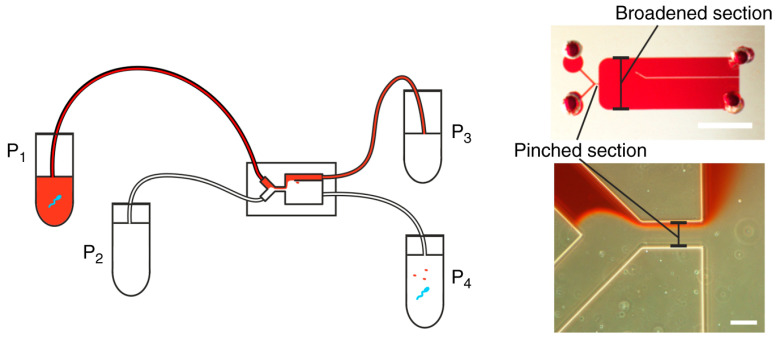
Pinched-flow fractionation (PFF) chip using a tumbling mechanism. The figure has been reproduced with permission from Springer Nature [69].

Berendsen et al. [69] proposed a microfluidic chip (Figure 1) based on the tumbling behavior of spermatozoa in pinched-flow fractionation which was used to separate spermatozoa from erythrocytes. Their study demonstrated a high extraction efficiency of 95% spermatozoa from a sample containing 2.5% spermatozoa while removing around 90% of the erythrocytes. Maenaka et al. [70] examined the availability of PFF for monodisperse droplets generated at the upstream T-junction via high-speed imaging. They reported a microfluidic system for continuous and size-dependent separation of droplets utilizing microscale hydrodynamics, which would be difficult for normal-scale schemes, such as centrifugation or filtration. Morijiri et al. [71] developed a microfluidic system based on the sedimentation effect of PFF, utilizing the inertial force of particle movement induced by the momentum change in the curved microchannel and the centrifugal force exerted on the flowing particles. In the study of Sai et al. [72], tunable pinched-flow fractionation (tunable PFF) was proposed as a modification of PFF with the introduction of a microvalve, where the effluent positions of the target particles can be controlled independently of the microchannel structure, which succeeded in separation micron and submicron-size polymer particles. Vig et al. [73] proposed a method for enhancing the separation of seven different polystyrene bead diameters ranging from 0.25 μm to 2.5 μm in PFF devices by a serpentine structure in the broadened segment, and the results demonstrated an amplification in the separation of up to 70%. Among the current microfluidic separation approaches, PFF is a cost-effective choice because of the simplicity of the device. However, there is a restriction for this method when vortices occur after the pinched segment with high Reynolds number (Re >> 1).

### 2.2. Inertia and Dean Flow

In fluid dynamics, secondary flow is a flow pattern, which is relatively weaker than the primary flow. The secondary flow can be controlled by the fluidic forces and the shape, size, and position of inserts [74]. In the study of macroscopic rigid spheres in Poiseuille flow by Segre and Silberberg particles migrated away from the wall and then accumulated at an equilibrium position of 0.6 from the axis around the tube radius due to lateral forces [75]. When a particle moves along a straight microchannel, two inertial lift forces are acting on the particle: shear-gradient-induced lift force, and wall-effect induced lift force [76]. Deformable particles contained in biomedical suspensions are underlying deformability-induced lift forces which lead to differences in dynamics [77]. The motion of a deformable particle in shear flow was studied by Bayareh and Mortazavi [78,79,80] with neglecting the gravity influence. Their results demonstrated that the equilibrium position of suspended particles is affected by the wall effect, deformability and sizes of particles, Reynolds number, density and viscosity ratio, etc. The nonlinear effects in finite-Reynolds-number flow were investigated, including the tubular pinch effect in cylindrical pipes [75]. Liu et al. [81] explored the focusing positions of different particle sizes in four focusing configurations for the separation of plasma, red blood cells, and cancer cells from the blood. The wall-induced inertia is significant in the thin layers near the walls where the lift is close to that calculated for linear shear flow, which increases dramatically with increasing Re above about 100 [82]. By analyzing the spatial distributions of spherical particles, Kim et al. [83] concluded the lateral migration of particles are induced by the high shear rate due to the small-scale effect and the particle equilibrium position as a function of Re. They observed the migration of particles markedly occurs at a very low Reynolds number and the critical Re when in the range of 20 to 30. Moreover, the inertial migration of spherical particles in a circular Poiseuille flow was numerically investigated with a Re smaller than 2200 [84]. A conclusion was drawn that the hydrodynamic interactions between the particles in different periodic cells have significant effects on the migration of the particles. The lateral migrations of viscous capsules [85], liquid drops, and vesicles [86] were also investigated.

Inertial microfluidics was applied in deformability-based cell classification and enrichment to reduce the complexity and costs of clinical applications [87]. Dean flow is a kind of secondary flow that can be generated by the fact that when a fluid flow in a curved pipe with a small radius of curvature, the flow has helical streamlines [88]. Focusing of particles suspended in solutions is largely independent of centrifugal forces, which suggests that Dean drag is the dominant lateral force to balance the influence of lift forces [89]. Di Carlo et al. [90] evaluated the migration attributed to lifting forces on particles in microfluidic devices by fabricating straight and curved microchannels under laminar flow conditions, when ordering is observed to be independent of particle buoyant direction. They developed a theoretical description of the underlying forces and a semiempirical relationship of cutoff and the channel geometry [91]. Inertia and Dean flow fractionation were applied in microfluidic separation and sorting of biochemical sample mixtures [40,75,92].

**Figure 2:**
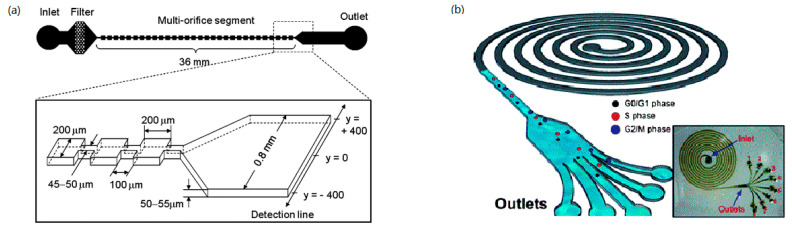
(**a**) Schematic diagram of multiorifice flow fractionation (MOFF) device. The figure has been reproduced with permission from the American Chemical Society [76]. (**b**) Schematic illustration of the spiral microfluidic design developed for cell-cycle synchronization. The figure has been reproduced with permission from the Royal Society of Chemistry [96].

The concept of inertial microfluidics was used in continuous separation of a multiparticle mixture in a simple spiral microchannel coupled with rotational Dean drag [93]. In inertial microfluidic experiments, the particle diameters cannot be very small compared to the characteristic channel length scale, and the Reynolds number of the particle is in order of 10 [94]. A spiral lab-on-a-chip (LOC) was used for size-dependent focusing of particles at distinct equilibrium positions across the microchannel cross-section from a multiparticle mixture [95], which exhibited 90% separation efficiency. Lee at al. [96] developed a spiral microchannel system for the synchronization and selection of cancer cells at different phases of cell cycle of blood to predict the condition of disease as shown in Figure 2b. Yousuff et al. [97] proposed a new configuration of spiral channel, where collection outlets are a series of side-branching channels perpendicular to the main channel of egress in which closely spaced particle streams can be collected separately. A novel inertial separation technique using spiral microchannel having a stair-like cross-section was introduced for size-based particle separation [98]. A spiral microfluidic chip was also employed for continuous separation of CTCs [99] and sperm-like-particles (SLPs) [100] from blood.

The secondary flow induced by a microchannel with arc-shaped groove arrays was studied by Zhao et al. [101] with numerical approaches, and their results showed the secondary flow can guide different-size particles to the corresponding equilibrium positions. In the experiments, the performance of particles focusing was relatively insensitive to the variation of flow rate, which proves the availability of flow-insensitive microfluidic separation method in a reliable biosample preparation processing step for downstream bioassays. Yoon et al. [95] developed a size-selective separation system for microbeads by using secondary flow induced by centrifugal effects in a curved rectangular microchannel. The effects of curvature angles and channel heights on inertial focusing of microparticles in curvilinear microchannels were also investigated by Özbey et al. [102], and an optimum condition/configuration was obtained with a curvature angle of 280° at Re of 144 in the transition region.

Inertial size separation can be achieved in a contraction–expansion array (CEA) microchannel by a force balance between inertial lift and Dean drag forces in fluid regimes in which inertial fluid effects are significant [103]. In CEA systems, similar effects compared to Dean flows are produced by an abrupt change of the cross-sectional area, which is balanced by inertial lift forces throughout the contraction regions [81]. The CEA microchannels are applied for high-yield blood plasma separation with a level of 62.2% yield [104]. A fishbone-shaped microchannel was proposed by Kwak et al. [105] to separate platelets, erythrocytes, and leukocytes from human blood.

Sim et al. [76] developed a novel separation method named as multiorifice flow fractionation (MOFF), where a microparticle moves laterally driven by the hydrodynamic inertial forces due to a multiorifice structure (Figure 2a). To improve the low efficiency of single-stage multiorifice flow fractionation (SS-MOFF) in separation for large particles, multistage multiorifice flow fractionation (MS-MOFF) was developed to isolate rare cells from human blood with a recovery increased from 73.2% to 88.7% while the purity slightly decreased from 91.4% to 89.1% [106]. A parallel multiorifice flow fractionation (p-MOFF) chip was developed and used for high-throughput size-based CTC separation, where CTCs can be focused at the center of the channel due to the wall-effect-induced lift force [107].

Separation of suspension in symmetric and asymmetric serpentine microchannels is also driven by inertial and Dean effects. Yuan et al. [108] investigated particle focusing under Dean flow coupled with elasto-inertial effects in symmetric serpentine microchannels, which demonstrated acceleration of particle focusing and reduction of channel length.

Compared with PFF, techniques based on inertia and Dean flow can be applied in higher Reynolds number flow since they are based on the balance of inertial shear-gradient-induced lift force and wall-effect-induced lift force, where the Reynolds number is generally in the range of 10–270 [34].

### 2.3. Deterministic Lateral Displacement (DLD)

**Figure 3:**
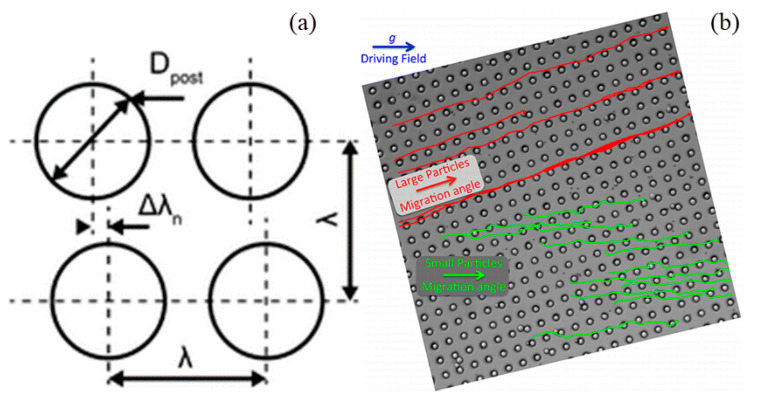
(**a**) Schematic of deterministic lateral displacement (DLD) chip with post placed at an angle to the flow direction. The figure has been reproduced with permission from the Royal Society of Chemistry [112]. (**b**) Microscopic image of gravity-driven deterministic lateral displacement (g-DLD) device. The figure has been reproduced with permission from the American Chemical Society [126].

Deterministic lateral displacement (DLD) is a microfluidic particle-separation device with asymmetric bifurcation of laminar flow around obstacles. When particles in solution moving through an array of obstacles, their paths are determined based on their sizes and deformability. The lateral displacement can be accumulated by a periodically arranged obstacle array which lead to a macroscopic change in migration angle, thus realizing particle separation [109]. Frechette et al. [110] used Stokesian dynamics simulation to study the dynamics of non-Brownian spheres suspended in a quiescent fluid and moving through a periodic array of solid obstacles under the action of a constant external force. It was found that moving particles were locked into periodic trajectories with an average orientation that coincides with one of the lattice directions. Generally, the arrangement of obstacle array has two configurations: a square array [111] and rhombic array obstacles (Figure 3a) [112]. The critical particle size for fractionation was investigated by Inglis et al. [113] who built a model based on the micropost geometry, where the fluid is driven by hydrodynamics or by electro-osmosis.

The fraction of whole-blood components and extraction of blood plasma without dilution was achieved by a continuous-flow deterministic array without dilution [114,115]. Blood components including white blood cells, red blood cells, and platelets can be separated by their hydrodynamic diameters from blood plasma at flow velocities of 1000 μm/s and volume rates up to 1 μm/min. A disposable parallel DLD device was applied for enrichment of leukocytes from blood with a throughput of greater than 1 mL/min [116]. With the utilization of an array of triangular instead of circular posts, the performance of DLD devices can be improved by reducing clogging, lowering hydrostatic pressure requirements, and increasing the range of displacement characteristics [117]. The DLD arrays with other shapes were investigated, including triangle [117], airfoil [118], I-shaped [119], L-shaped [120], asymmetric shape [121], and optimized shape [122], which have been used instead of cylindrical one. The elastomeric properties of PDMS were utilized to achieve tunable particle separation in DLD devices [123]. With the introduction of an external force, a concept of force-driven DLD was proposed [124]. For overdamped particles under the action of external forces, the trajectories are periodic, and the migration angle corresponds to a tangent bifurcation [125]. Devendra et al. [126] investigated the continuous size-based separation of suspended particles in gravity-driven deterministic lateral displacement (g-DLD) devices. (Figure 3b) In their experiments, directional locking angles were strongly depended on the size of the particle, and the results suggested that relatively small forcing angles are well suited for size-fractionation purposes. In an upscaled DLD device, larger gaps were utilized instead of micrometer-sized gaps between the posts, where particles above a critical size were better separated [127].

DLD devices are also employed for the separation of CTCs [128], sleeping parasites [111], and deformable particles [129,130,131] by applying different pressures to the flowing fluids. A novel method for passive separation of microfluidic droplets by size using DLD was proposed by Joensson et al. [132], which showed a rate of 12,000 droplets/s with an 11 μm diameter. DLD separation for droplets can be accelerated by cell-induced shrinking [133]. A microfluidic DLD device was applied for spore purification to reduce the amount of debris in a suspension of fungal spores with almost 100% purity and recovery in continuously microspheres [134]. DLD techniques are suitable for the sorting of kinds of biological particles and droplets, but such a method requires an array of posts.

### 2.4. Microscale Filters

Microscale filters are widely employed in the separation of bioparticles/droplets based on size and deformability [40]. The most commonly used types of microfilters are categorized as dead-end mode [135], where the low is perpendicular to the filter structure, including membrane [136], planar [137], weir [138], pillar [139], and crossflow filters [114], where the flow is in the direction of the filter plane [140].

**Figure 4:**
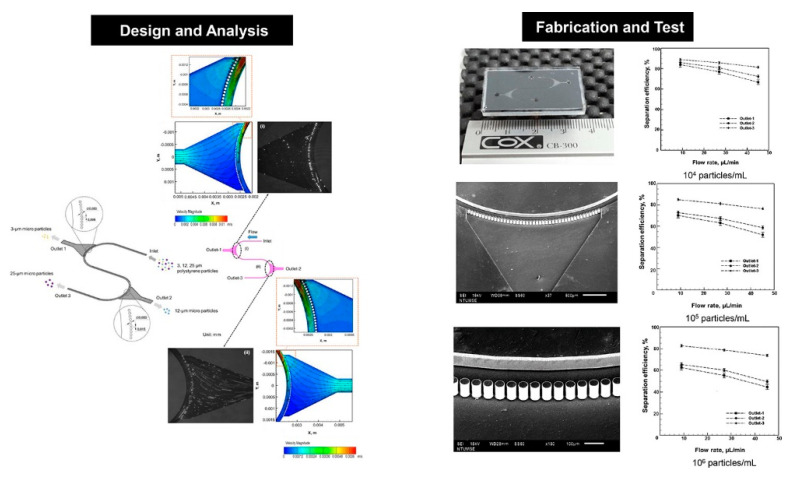
Crossflow microfilter with multistage dual arc-unit structures. The figure has been reproduced with permission from Elsevier [141].

Membrane-based separation is a pressure-driven process [141,142], which has been widely used for microfiltration, ultrafiltration, reverse osmosis, ion-exchange, and gas separation [143]. The size-based crossflow separation can also be achieved using multistage arc-unit structures in a microfluidic device as shown in Figure 4 [141]. Chen et al. [144] proposed a method for preparation of microfiltration membranes made up with cellulose acetate (CA) blended with polyethyleneimine (PEI), where PEI can provide coupling sites for ligands in affinity separation or be used as a ligand for metal chelating, endotoxin removal, or ion exchange. In the study of Aussawasathien et al. [145], electrospun nylon-6 nanofibrous membranes were employed as prefilters for separation of micron to submicron particles from water due to their excellent chemical and thermal resistance as well as high wettability. A PDMS-membrane microfluidic immunosensor was used for rapid detection of foodborne pathogens integrated with a specific antibody-immobilized alumina nanoporous membrane. By sandwiching a filter membrane between a two-layer chip, Liu et al. [146] developed a vacuum-accelerated microfluidic immunoassay (VAMI), which could simultaneously achieve higher sensitivity and require less time compared with conventional microfluidic immunoassays. Nam et al. [136] proposed a novel effective manufacturing process that uses reusable 3D silicon molds with microneedle and microblade shapes to form submicron-sized nanopores and slit arrays in PDMS films. This process has been successfully applied to trap submicron-sized bacteria with a filter recovery rate of 90.1%. A superhydrophilic membrane with rough and hierarchical structures was used in the separation of oil-in-water emulsions since it can be fouled by surfactant-stabilized oil and organic foulants [147]. Ng et al. [148] designed and fabricated different gradient ceramic membranes including one-, two-, and three-layer ceramic membranes with a low total resistance, which demonstrated that the gradient porous membrane can be used to enhance the filtration performance.

Besides membranes, various types of microfabricated filters have been developed for microparticle separation. Crowley et al. [137] developed a planar microfilter for the isolation of plasma from whole blood with a separation efficiency three times higher than microporous membranes. An array of micropillars with a diameter of 12 μm and a height of 15 μm was arranged in I-shape as a filter for the separation of spherical and nonspherical particles [139]. Compared with pillar type, the microfilters of weir type show a higher separation efficiency due to the small gap of pillar [149]. A slanted weir microfluidic device was applied for the separation of CTCs from the peripheral blood, which showed a 97% separation efficiency as well as an 8-log depletion of erythrocytes and 5.6-log depletion of leukocytes [138]. As a modification, a cascading weir-type microfilter was constructed by Wu et al. [140] for plasma separation from blood samples.

**Figure 5:**
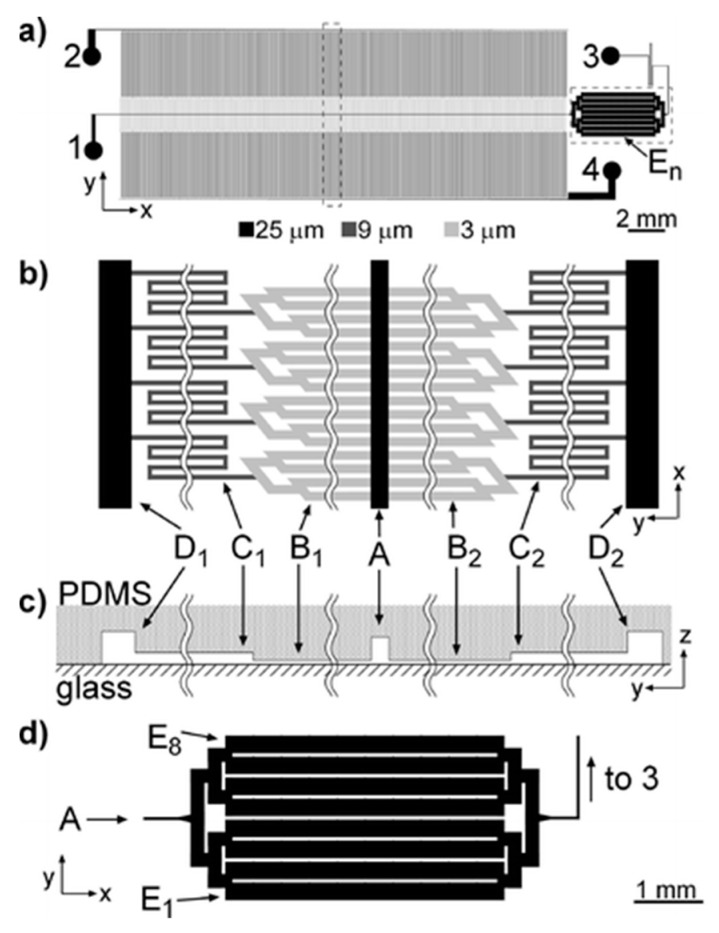
(**a**) Drawing of the microfluidic device, ports labeled 1–4 are blood inlet, perfusion inlet, WBC outlet, and RBC outlet, respectively; (**b**) blowup of a fragment of the separation network outlined with a dotted line in (**a**) turned counterclockwise by 90° with respect to (**a**); (**c**) cross-sectional view of channels in the separation network, dimensions are not to scale; (**d**) blowup of E channels outlined with a dotted line in (**a**). Channel depths, 25, 9, and 3 μm, are grayscale coded in (**a**,**b**,**d**). The figure has been reproduced with permission from the American Chemical Society [151].

The separation of microparticles was reported to be achieved in crossflow microfilters for cell biology research or various diagnostic and therapeutic applications, including cells extraction [150,151,152], plasma fabrication [137,153], leukapheresis [154], and myocytes/nonmyocytes from neonatal rat myocardium [155]. A microfluidic technique was proposed for separation of white blood cells (WBCs) from whole human blood, where the separation was performed in crossflow in an array of microchannels with a deep main channel and a large number of orthogonal and shallow side channels [151], as shown in Figure 5. The flow and shear stress characteristics inside a crossflow filter were studies by Mielink et al. [156] with employing microparticle image velocimetry (micro-PIV) measurements and computational fluid dynamics (CFD) analysis, demonstrating filter performance can be improved since substantial increase in the local wall shear can reduce clogging and cell cake formation.

Moorthy et al. [157] proposed in situ fabrication of porous filters using emulsion photopolymerization for microsystems to mimic the functionality of the centrifuge and power requirements as well as enabling the handling of small sample volumes. A novel microfluidic device constituted by microfilter, micromixer, micropillar array, microweir, microchannel, and microchamber was fabricated and used for isolation of WBCs from RBCs of whole blood [152]. Aran et al. [158] developed a microfiltration system consisted of a two-compartment mass exchanger with two aligned sets of PDMS microchannels, separated by a porous polycarbonate (PCTE) membrane. Lo et al. [159] described a multichamber device with porous membranes incorporated with variable pore sizes between the compartments within the microfluidic device, where nonhomogenous cell mixtures can be fractionated into different compartments in stages and collected for further analysis.

### 2.5. Other Hydrodynamic Methods

**Figure 6:**
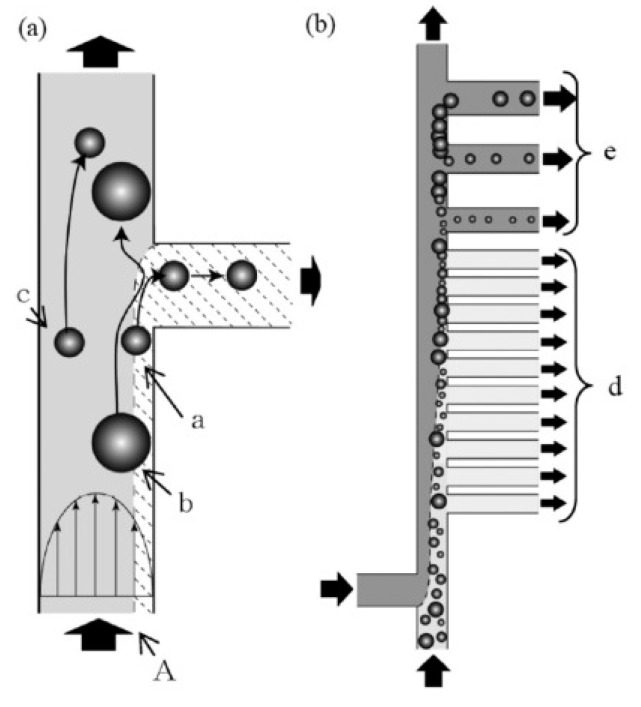
Principle of particle classification and concentration: (**a**) particle behavior at a branch point; (**b**) schematic diagram of particle classification and concentration in microchannel having multiple branch points and side channels. The figure has been reproduced with permission from John Wiley and Sons [161]. “a” represents a particle can enter the side channel, “b” and “c” represent particles that cannot enter the side channel; “d” represents the area where particles larger than a certain size cannot pass through; “e” represents the downstream area where particles are removed from the main stream in ascending order of size. A is the borderline and when a particle flows in the right region of the borderline, such a particle can enter the side channel.

Besides the methods listed above, other hydrodynamic methods are also explored to be employed in separation of microparticles, including hydrodynamic filtration [160,161,162,163], Zweifach–Fung effect [164,165,166,167,168,169], trilobite separator [170,171,172,173,174], microvortex [175], and microhydrocyclone [176]. For particles flowing in a microchannel, their center positions cannot be at a certain position where the distance from sidewalls is equal to the particle radius. Yamada et al. [160] proposed the method of hydrodynamic filtration (HDF) for continuous concentration and classification of particles within microfluidic devices. By withdrawing a small amount of liquid repeatedly from the main streams through the side channels, particles are concentrated and arranged on the sidewalls by repeatedly drawing a small amount of liquid from the main flow through the side channel. Then, the concentrated and arranged particles can be collected through other side channels in downstream according to their sizes. Therefore, continuous introduction of the particle suspension into the microchannel can simultaneously perform particle concentration and classification. In this method, the flow profile inside the precisely manufactured microchannel determines the size limit of the filtered materials. Thus, the separation for small particles in much larger channels avoiding the problem of channel clogging. This device was applied for blood cell classification [161], as shown in Figure 6, and the sorting efficiency of hydrodynamic filtration device was dramatically improved by employing a flow splitting and recombination scheme [162]. Chiu et al. [163] proposed a microfluidic chip to separate microparticles using crossflow filtration enhanced with hydrodynamic focusing, which is needed to make soft lithograph fabrication to create microchannels and uses novel pressure bonding technology to make high-aspect-ratio filter structures.

Zweifach–Fung effect was the principle that a particle tends to follow the high-flow-rate channel when it reaches a bifurcation region [164]. This effect was employed for the separation of RBCs from plasma [165] and whole blood [166] and bacteria from blood [167]. The suspension stability of the blood was investigated by Fahraeus [168], and aggregation was observed to occur at a high concentration of blood under the influence of gravity and surface charge. Based on the characteristics of blood, Geng et al. [169] developed a device for separation of plasma from whole blood using a combination of Zweifach–Fung bifurcation law, centrifugation, and diffuser–nozzle effect.

Sample concentration or enrichment for rare particles in centrifugal separator often results in the cell being crushed and congregated during processing. Aiming to develop a nonclogging microconcentrator, Dong et al. [170] proposed a trilobite microchip for CaSki cells concentration using streamlined turbine blade-like micropillars based on the counter-flow principle. Hønsvall et al. [171] developed a microfluidic chip for continuously concentrating rigid cells in moving fluids based on a trilobite structure, which appears to be a promising tool for preconcentrating microalgae that are difficult to harvest due to their repelling properties or small size. The separation and concentration characteristics of the so-called trilobite separation unit was characterized experimentally by Mossige et al. [172]. With the introduction of a tunable structure, an increase in flow rate for low-pressure drops can be realized thus enabling clog-free particle separation of complex algal cells [173,174]. Besides the methods above, microvortex manipulator (MVM) [175] and microhydrocyclone [176] are also categorized as hydrodynamic methods for microfluidic separation and focusing of particles. The major public-health-related microfluidic separation/sorting technologies working in a passive way are summarized in Table 1.

## 3. Microfluidic Detection Methods

Microfluidic-method-integrated detection equipment has been becoming an ideal portable device for field sampling. Moreover, it improves the efficiency, sensitivity, and accuracy of detection and has advantages of rapid analysis, less usage of sample, and real-time characterization. Herein, microfluidic-based detection methods were summarized, including electrochemical detection, optical detection, and magnetic detection.

### 3.1. Electrochemical Detection

Electrochemical methods have advantages of shorter testing time, simpler device, and low cost, which can be classified into amperometric detection [177,178], impedimetric detection [179,180], and potentiometric detection [181]. Amperometric detection was formed when electroactive substances or electrolytes containing ions are under the action of an electric field, and they can be separated and detected effectively. Shiddiky et al. proposed an electrochemical detection method combined with micellar electrokinetic chromatography to separate and detect trace phenolic compounds in water [182]. They first used field-amplified sample stacking (FASS) and field-amplified sample injection (FASI) to separate the samples from water and then used cellulose-double-stranded DNA modified screen-printed carbon electrode to amplify the electrooxidation sensitivity of eight phenolic compounds. Hiraiwa et al. developed a method that used microtip immunoassay to detect the *Mycobacterium tuberculosis* (MTB) in sputum [183]. The microtip coated by antibodies was used to capture targeted bacteria. After that, the microtip surface would be covered by immunocomplex which can be detected by electric current. The detection limit of this method was 100 CFU per milliliter.

**Figure 7:**
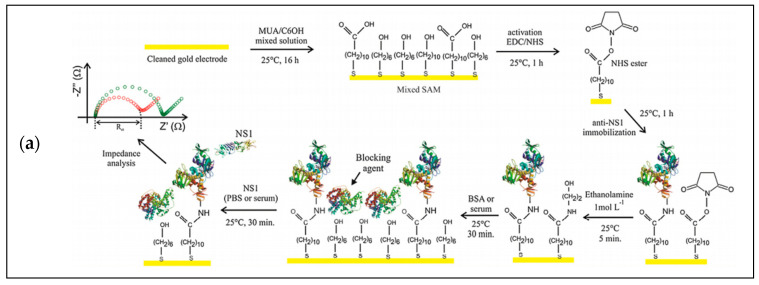

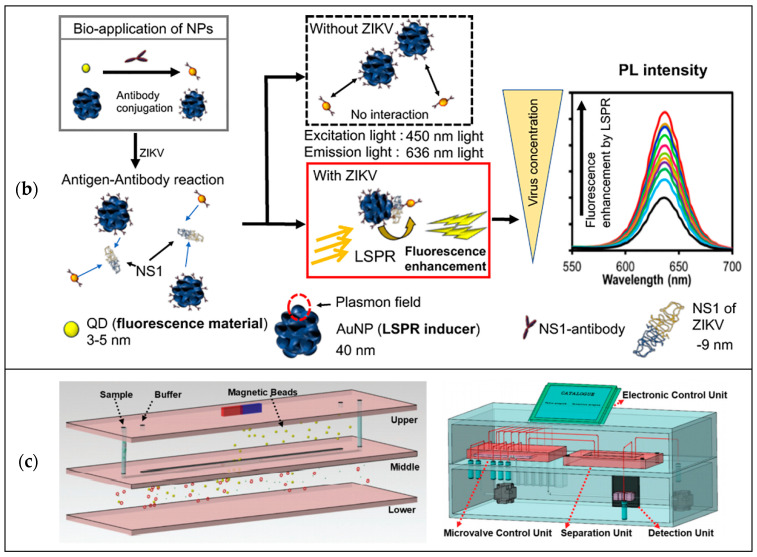
(**a**) Steps of electrode functionalization of the impedimetric biosensor to test neat serum for dengue diagnosis. The figure has been reproduced with permission from Elsevier [185]. (**b**) Schematic representation of the localized surface plasmon resonance (LSPR)-amplified immunofluorescence biosensor. The figure has been reproduced with permission from Takemura et al. [191]. (**c**) Schematic of the detection device based on the 3-D magnetophoretic separation and magnetic label. The figure has been reproduced with permission from the American Chemical Society [200].

Impedimetric detection is a method using electrochemical impedance spectroscopy (EIS) for analysis. It has merits of the advantages of label-free and less amplitude disturbance [184]. As shown in Figure 7a, Cecchetto et al. proposed a label-free impedimetric detection method with a gold electrode modified by an anti-NS1 and a nonstructural dengue protein antibody to diagnose the dengue by detecting neat serum through the resistance changes resulting from the target binding [185].

Potentiometric detection is based on the potential change in an electrode in an electrochemical cell. The advantages of potentiometric biosensors are small volume, fast response, easy to use, low cost, anticolor, antiturbidity interference, and independent of sample volume [186,187]. For example, an electrochemical paper-based analytical device (EPAD) was designed to measure the concentrations of electrolyte ions (Cl−, K+, Na+, and Ca2+). In this design, ions were able to across the paper channels slowly so that accuracy was improved [188].

### 3.2. Optical Detection

Optical detection utilizes the properties of light, such as absorbance, fluorescence, and the emission mode of the sample when excited. Among optical detection methods, the fluorescence method is commonly used because it is sensitive, cheap, fast, and easy to operate [189]. The key to designing a fluorescence biosensor is fluorescent dyes or the labeling of fluorophores. Using fluorescence resonance energy transfer (FRET) is one of the most typical strategies, referring to the energy transfer from a donor fluorophore to an acceptor fluorophore [190]. Moreover, some nanomaterials also have fluorescence signals under specific conditions base on their unique properties of physical, chemical, and electronic transport. As shown in Figure 7b, Takemura et al. [191] designed an optical detection method using quantum-dots-based immunofluorescence to detect nonstructural protein 1 (NS1) of Zika virus. The fluorescence intensity signal was amplified and detected by a localized surface plasmon resonance (LSPR) signal from plasmonic gold nanoparticles (AuNPs). This sensor can detect NS1 of Zika virus ultrasensitively, rapidly, and quantitatively. In addition to the fluorescence method, absorbance of samples can be used to realize target analysis. For example, the analysis of UV absorption of nitrite samples can be used to determine the nitrite level in water [192].

Recently, surface-enhanced Raman scattering (SERS) spectroscopy has advantages of strong signal intensity, excellent photostability, biocompatibility, and especially the multiplexing ability, which makes it become a popular optical imaging and detection tool. For example, Wang et al. [193] first used folic acid (FA) functionalized gold (Au) SERS nanoparticles to detect CTCs in the presence of white blood cells successfully. Wu and co-workers have improved the sensitivity and specificity of CTC detection using the SERS properties of gold or silver with various shapes [194]. Moreover, Quang et al. [195] successfully demonstrated that the portable Raman spectrometer can be used to detect dipicolinic acid (DPA) and malachite green (MG) in real time, combined with a micropillar array chip.

### 3.3. Magnetic Detection

In the past few decades, the magnetic phenomenon of magnetic materials has been widely concerned, which is used to realize the sensitive detection of analytes [196]. Compared to the optical detection method, the magnetic detection method has advantages of low cost and high detection efficiency because of the elimination of expensive optical elements and the use of a magnetic field to shorten the sample preparation time [197,198]. Moreover, because biological samples have few magnetic background signals which can be ignored, the magnetic detection method has high specificity, sensitivity, and signal-to-noise ratio [199]. Hong et al. constructed an automated detection device for H7N9 influenza virus hemagglutinin, assisted by three-dimensional (3-D) magnetophoretic separation and magnetic label [200]. As shown in Figure 7c, a 3-D microchannel network with two-level channels was generated with multilayer glass slides under a magnetic field perpendicular to the microchannel. After the immunomagnetic separation, a magnetic-tagged complex was captured by an antibody-modified glass capillary, which causes the change of voltage in the miniature tube liquid sensor and therefore to obtain the detection signal. This work achieved the detection limit of 8.4 ng mL^−1^ for H_7_N_9_ hemagglutinin, with good specificity and reproducibility. Wu et al. [201] reported a Z-Lab point-of-care (POC) device which can detect swine influenza viruses sensitively and specifically reducing the dependence on the demands of sample treatment and operational skills sample handling and laboratory skill requirements. In this work, a portable and quantitative, giant magnetoresistive (GMR)-based immunoassay platform was designed to detect IAV nucleoprotein (NP) and purified H_3_N_2_v. It can achieve quantitative results within 10 min with a detection limitation of 15 ng per milliliter for IAV nucleoprotein, and 125 TCID50 per milliliter for purified H_3_N_2_v. Wu et al. [202] also introduced a new magnetic particle spectroscopy (MPS)-based biosensing scheme, where self-assembly magnetic nanoparticles (MNPs) can be used to detect H_1_N_1_ nucleoprotein molecules quantitatively. This work verified that it is reliable to use MPS and the self-assembly of MNPs to detect ultralow concentrations of targeted biomolecules, which can be applied on rapid, sensitive, and wash-free magnetic immunoassays.

Although these detection methods have good performance, they still have many shortcomings [203]. For electrochemical detection methods, they have high sensitivity, fast response, and low cost, but stability and susceptibility to interference are weak [204]. Optical detection methods have advantages of rapid response, flexibility, and experimental simplicity, but they are impacted by a high fluorescence background and short fluorescence lifetime [205]. Magnetic detection methods have advantages of low cost, high detection efficiency, high specificity, sensitivity, and signal-to-noise ratio but are limited by a shortage of miniaturized magnetic readout systems [206].

## 4. Prospects of Microfluidics for Public Health Applications

In this paper, the emerging microfluidics studies for separation and detection have been overviewed, which have been widely applied in public health. In the context of an epidemic of infectious diseases, point-of-care diagnostics have become a matter of great concern, which enable people to implement home quarantine and real-time health monitoring. This method can cut off the source of infection and thus greatly reduce the rate of infection rate. Meanwhile, the fast development of microfluidics in the field of medicine enables point-of-care diagnostics to be realized. As mentioned above, microfluidics has advantages of less sample consumption, fast detection speed, facile operation, multifunctional integration, lower cost, and portability. The employment of microfluidic devices combined with point-of-care diagnostics can reduce the cost of public health care. Microfluidics can be well applied on virus detection, for example COVID-19 diagnosis. COVID-19 can be detected from saliva and respiratory samples of nasopharyngeal and oropharyngeal swabs by quantitative reverse-transcription polymerase chain reaction (qRT-PCR). COVID-19 can be identified through the variations of many biomarkers such as immunoglobulins, cytokines, and nucleic acids. Fast and accurate detection of these biomarkers by microfluidic system can be helpful in early diagnosis of COVID-19. Moreover, a microfluidic system combined with smartphones may realize the real-time health monitoring of individuals or populations during and after COVID-19 outbreaks. However, some detections such as impedance-based microfluidic devices and optical microfluidic devices require bulky instrumentation for the quantification of results. Besides, most samples require multiple pretreatments before detection. Therefore, although microfluidics combined with point-of-care diagnostics have the potential to allow the rapid detection of COVID-19 or other diseases, there is still a gap to be bridged.

Microfluidic techniques can also be applied in continuous production of vaccines [207]. For instance, the range of technology platforms for COVID-19 vaccines includes nucleic acid (DNA and RNA), virus-like particle, peptide, viral vector (replicating and nonreplicating), recombinant protein, live attenuated virus, and inactivated virus approaches, where microfluidic approaches can be applied [208,209,210]. Microfluidic devices were employed for vaccine therapy and delivery, especially for the administration of nucleic-acid-based vaccines by employing the host cell’s transcriptional and translational capability to produce the desired protein, since uniform microspheres of DNA/RNA with a very narrow size distribution can be produced precisely [211]. Compared with other kinds of vaccines, because the vaccines of DNA or RNA do not have a viral coating, there is no requirement to invoke antibody reactions in order to suppress vaccine efficiency. Moreover, such vaccines are safe and easy to produce, thus presenting the opportunity for combining the genetic information of various antigen epitopes and cytokines [212].

## 5. Conclusions

Dramatic growth in microfluidic and lab-on-a-chip technologies has paved a way for the development of appropriate separation and detection-based diagnostics with the goal of improving local and global public health and thereby has attracted considerable efforts and resources in the past decade. Access to effective and efficient separation and detection methods has become increasingly important especially during the pandemic period. However, there exist several key factors that affect the introduction, acceptability, and sustainability of these technologies for practical applications; one of the greater challenges in deploying microfluidic diagnostic systems on a larger scale and to a wider extent is how to bring the cost down closer to the cost of the most inexpensive of current tests. The second challenge is that the performance of these methods is not good enough and needs to be further improved. This can be achieved by using a multistep method, which may lead to higher particle or cell separation performance. At the same time, a multistep method requires complicated configuration and a higher level of automation and integration technology. In addition, the production capacity of microfluids is far from meeting the actual needs. By increasing the number of devices running in parallel or the number of separation or detection units in the same microfluidic system, it is inevitable to enlarge the microfluidic technology. Accuracy and repeatability are also very crucial, and it is expected that an automated apparatus should be used as much as possible without much intervention from human operators. More sustainable efforts are required in the future to apply microfluidic technologies in developing more effective clinical or point-of-care tools, as well as detection systems to monitor the environmental conditions.

## Figures and Tables

**Table 1:** Public-health-related passive approaches for microfluidic separation.

Categories	Examples	References
Pinched-flow fractionation (PFF)	Symmetric PFF	[65]
	AsPFF	[67,68,70,73]
	Tumbling mechanism in PFF	[66]
	Sedimentation PFF	[71]
	Tunable PFF	[72]
Inertia and Dean flow	Inertial and Dean flow fractionation	[40,79,89,93]
	Spiral microchannel	[97,98,99,100]
	Curvature angles	[90,101,102]
	CEA	[83,103,104,105]
	Multiorifice	[66,106,107]
	Serpentine microchannel	[108]
Deterministic lateral displacement (DLD)	DLD	[109,110,111,112,113,114,115,127,128,129,130,131,134]
	Disposable parallel DLD	[116]
	Optimized shape	[117,118,119,120,121,122]
	Tunable DLD	[123]
	Force-driven DLD	[124,125,126]
	Droplet shrinking	[132,133]
	Membrane	[136,144,145,147,148]
Microscale filter	Vacuum-accelerated microfluidic immunoassay (VAMI)	[146]
	Planar microfilter	[137,139]
	Weir microfluidic device	[138,140]
	Crossflow microfilter	[150,151,152,153,154,155,156]
	Porous filter	[156,158]
	Multicompartment	[159]
Other hydrodynamic methods	Hydrodynamic filtration	[160,161,162,163]
	Zweifach–Fung effect	[164,165,166,167,168,169]
	Trilobite separator	[170,171,172,173,174]
	Microvortex	[175]
	Microhydrocyclone	[176]
